# Inactive and sedentary lifestyles amongst ambulatory adolescents and young adults with cerebral palsy

**DOI:** 10.1186/1743-0003-11-49

**Published:** 2014-04-03

**Authors:** Carla FJ Nooijen, Jorrit Slaman, Henk J Stam, Marij E Roebroeck, Rita J van den Berg-Emons

**Affiliations:** 1 Department of Rehabilitation Medicine, Research lines MoveFit & Transition and Lifespan Research, Erasmus MC, University Medical Centre, P.O. Box 2040, 3000 CA Rotterdam, The Netherlands

**Keywords:** Physical behaviour, Cerebral palsy, Sedentary time, Physical activity, Accelerometer

## Abstract

**Background:**

To assess physical behaviour, including physical activity and sedentary behaviour, of ambulatory adolescents and young adults with cerebral palsy (CP). We compared participant physical behaviour to that of able-bodied persons and assessed differences related to Gross Motor Functioning Classification System (GMFCS) level and CP distribution (unilateral/bilateral).

**Methods:**

In 48 ambulatory persons aged 16 to 24 years with spastic CP and in 32 able-bodied controls, physical behaviour was objectively determined with an accelerometer-based activity monitor. Total duration, intensity and type of physical activity were assessed and sedentary time was determined (lying and sitting). Furthermore, distribution of walking bouts and sitting bouts was specified.

**Results:**

Adolescents and young adults with CP spent 8.6% of 24 hours physically active and 79.5% sedentary, corresponding with respectively 123 minutes and 1147 minutes per 24 hours. Compared to able-bodied controls, persons with CP participated 48 minutes less in physical activities (p < 0.01) and spent 80 minutes more sedentary per 24 hours (p < 0.01). Physical behaviour was not different between persons with GMFCS level I and II and only number of short sitting bouts were significantly more prevalent in persons with bilateral CP compared to unilateral CP (p < 0.05).

**Conclusions:**

Ambulatory adolescents and young adults with CP are less physically active and spend more time sedentary compared to able-bodied persons, suggesting that this group may be at increased risk for health problems related to less favourable physical behaviour.

**Trial registration:**

Nederlands trial register: NTR1785

## Background

Physical activity has been defined as “any bodily movement that results in energy expenditure” [1]. Physical activity contributes to the primary and secondary prevention of several chronic diseases, including cardiovascular disease, cancer, diabetes mellitus, hypertension and obesity, and is associated with a reduced risk of premature death in the general population [2]. Sedentary behaviour, defined as a distinct class of activities that require low levels of energy expenditure and involve sitting and lying [3], also negatively impacts metabolism and cardiovascular health [4]. Physical activity and sedentary behaviour are distinct aspects of physical behaviour [5]. Independent of physical activity, a person with a large amount of sedentary time may still be at risk of poor health outcomes [4]. Consequently, besides meeting physical activity guidelines it is also recommended to limit the amount of sedentary time [4].

Persons with cerebral palsy (CP) experience problems with movement and posture, including difficulty with balance and walking, gross and fine motor control, and muscle spasticity. Therefore, they are at risk of reduced physical activity and increased sedentary behaviour [6]. Previously, it has been indicated that children and adults with CP participate substantially less in physical activities compared to reference populations, and less than recommended by guidelines [7-9]. With regard to sedentary behaviour, children aged 5 to 17 years with CP fail to achieve recommended activity levels [7]. To our knowledge, sedentary behaviour has not been studied previously in persons with CP after childhood.

Transition to adulthood is thought to be an important time for interventions that promote physical activity and limit sedentary time because at this age many changes in life may influence the adult lifestyle [10,11]. However, to our knowledge, physical behaviour for 16 to 24 year-olds has not yet been studied in persons with CP. Knowledge of physical behaviour at this age can help optimise recommendations and treatments to increase physical activity and limit sedentary behaviour in persons with CP across the lifespan. Furthermore, by comparing physical behaviour of subgroups based on CP characteristics, recommendations and treatments can be further optimised and tailored for disorder severity.

Therefore, the aim of the current study was to assess physical behaviour of ambulatory adolescents and young adults, aged 16 to 24 years, with spastic CP. Physical behaviour variables included objectively measured physical activity and objectively measured sedentary behaviour. Total duration, intensity and types of physical activities (walking, running, cycling, and non-cyclic movement) were assessed, and distribution of walking bouts was described. Total sedentary time was determined (sitting and lying) and specified with regard to total duration of sitting and distribution of sitting bouts. Furthermore, self-reported physical activity was assessed. Objective data were compared with data of able-bodied controls, and differences within the CP group related to Gross Motor Functioning Classification System (GMFCS) and distribution of CP (unilateral/bilateral) were explored.

## Methods

This study is part of the longitudinal, multi-centre, randomised controlled trial Learn2Move 16–24, which evaluates an intervention to promote daily physical activity and sports participation, reduce sedentary behaviour, and improve physical fitness amongst adolescents and young adults with spastic CP [12]. In the current study, baseline data from the longitudinal study were used.

### Participants

Adolescents and young adults with spastic unilateral or bilateral CP, aged 16 to 24 years, were recruited from six rehabilitation centres and rehabilitation departments at university hospitals in west-central Netherlands, and by the Association of Physically Disabled Persons and their Parents.

Exclusion criteria were: 1) disabilities other than CP that affect physical activity or physical fitness; 2) contraindications to (maximal) exercise; 3) severe cognitive disorders or insufficient comprehension of Dutch; 4) partly dependent or fully dependent on a manual wheelchair; 5) physical activity level higher than 15.6% of 24 hours (mean physical activity level + 2 standard deviations (SD) of an adult CP population) [9]. No one was excluded by this latter criterion.

All participants provided written informed consent. The study was approved by the Medical Ethics Committee of the Erasmus Medical Centre. Local approval was granted by all participating centres.

### Physical behaviour

**Figure 1:**
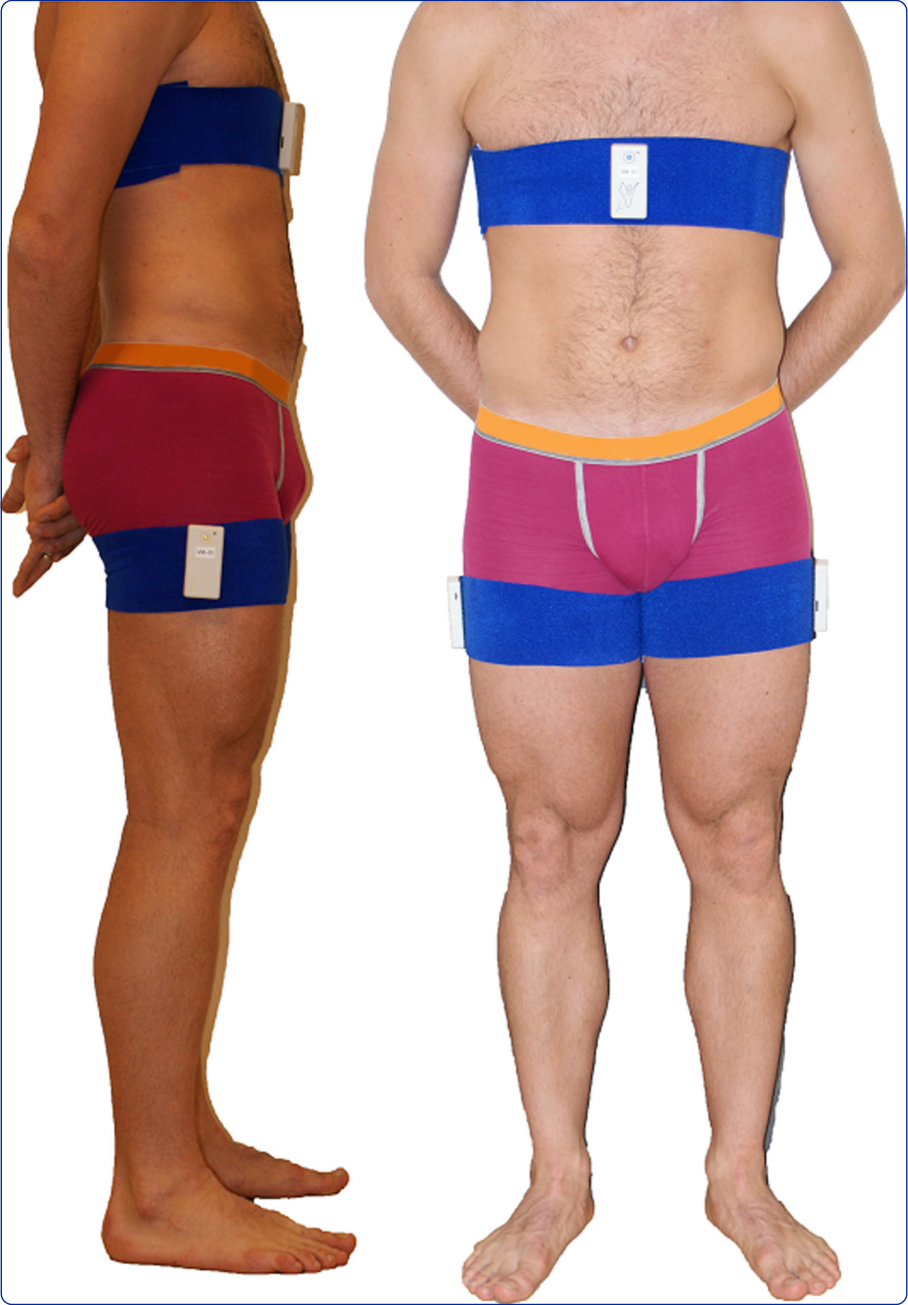
VitaMove activity monitor.

To objectively measure physical behaviour, we used the ambulatory monitoring system VitaMove (2M Engineering, Veldhoven, The Netherlands), with body-fixed accelerometers (Freescale MMA7260Q, Denver, USA) (Figure 1). This activity monitor has demonstrated validity to quantify mobility-related activities and postures and to detect intergroup differences in physical behaviour [13,14]. The system consists of three recorders that are wirelessly connected and synchronised every ten seconds. One recorder was attached to each thigh and a third recorder was attached to the sternum. The recorders were worn on the body using elastic belts. The measurements were started at participants’ homes and activity monitors were worn continuously on consecutive weekdays, except during swimming, bathing and sleeping. Participants kept activity diaries that allowed for correction for periods of non-wearing time of the activity monitor. The intended duration of measurement was 72 hours with a minimum duration of 24 hours. This minimum duration was previously established as adequate for determining activities and postures [15]. To avoid measurement bias, we instructed participants to continue their ordinary daily life and the principles of the activity monitor were only explained after study completion.

Accelerometer signals for each recorder were sampled and stored digitally on a micro Secure Digital memory card. Measurements were uploaded to a computer for kinematic analysis using VitaScore Software (VitaScore BV, Gemert, The Netherlands). The duration, rate, and moment of occurrence of physical activity, sedentary behaviour, and transitions between postures were automatically and separately detected with a 1-second resolution. Furthermore, motility was determined, which provides information on the variability of the acceleration signal and is related to the intensity of body-segment movements. A detailed description of the configuration and analysis has been described elsewhere [13].

The following data were obtained:

1. Total duration of physical activities, including walking, running, cycling, and non-cyclic movement and separate duration of each of these activities. All physical activity measures were expressed as a percentage of a 24-hour period.

2. Total duration of sedentary behaviour, including sitting and lying, and separate duration of sitting and standing, all expressed as a percentage of a 24-hour period.

3. Mean motility of the total of physical activities and mean motility of walking, expressed as gravitational force (g).

4. Distribution of continuous walking and sitting bouts with pre-defined durations: 0–10 sec; 10–60 sec; 1–10 min; 10–30 min; or > 30 min.

For reference, we used activity monitor data of 32 able-bodied persons aged 14 to 29 years available from previous studies at our department (mean age 22 years (SD = 5), 14 males). All able-bodied persons wore the activity monitor for two consecutive weekdays. Measurements were performed with a non-wireless version of the activity monitor and analysed with a previous software version. However, the underlying technique of the activity monitor is the same as that of the monitors worn by the participants with CP, and the algorithms of data analysis comparable between software versions. Data for participants with CP were expressed as a percentage of reference data.

Self-reported physical activity levels were measured with the Dutch version of the Physical Activity Scale for Individuals with Physical Disabilities (PASIPD) [16], a 13-item, 7-day recall questionnaire developed for people with a physical disability. The scale consists of questions regarding leisure time, and household-related and work-related physical activity. The total PASIPD score was calculated by multiplying the average hours per day for each item by a given metabolic equivalent (MET) value associated with the intensity of the activity. Because the PASIPD was developed for persons with physical disabilities, there are no reference data for able-bodied persons.

### Statistical analysis

An independent t-test was used to test for differences in age and a Chi-Square test to test for difference in gender between the total group of participants with CP and able-bodied persons. Regression analyses, correcting for age and gender, were used to assess differences in physical activity and sedentary time between participants with CP and able-bodied persons. Regression analyses correcting for age and gender were used to test for differences in physical behaviour between subgroups on basis of GMFCS level and CP distribution. Statistical analyses were performed using SPSS 20 (SPSS Inc, Chicago, IL). The significance level was set at p < 0.05.

## Results

In total, 48 ambulatory adolescents and young adults with CP completed the physical behaviour measurements. Due to technological challenges with the activity monitor, data were not available for the intended three days for all participants. Measurement duration was 72 hours for 37%, 48 hours for 51% and 24 hours for 12% of participants.

Characteristics of participants with CP and able-bodied persons are described in Table 1. No significant differences were found in age (p = 0.1) and gender (p = 0.5) between these groups. Furthermore, Table 1 shows data on physical activity, sedentary time and self-reported physical activity, and the comparison with able-bodied persons. Compared to able-bodied persons, persons with CP were significantly less physically active (p < 0.01) and spent more time sedentary (p < 0.01). On average, persons with CP participated 48 minutes per 24 hours less in physical activities compared to able-bodied controls (123 vs. 171 minutes/24 h). Sedentary time was 80 minutes per 24 hours more in the group with CP compared to able-bodied controls (1147 vs. 1077 minutes/24 h). Self-reported physical activity level in participants with CP was on average 13.0 (8.6) MET-hr/day.

**Table 1:** Characteristics, physical activity, sedentary time and self-reported physical activity

	**CP**	**Able-bodied**^**4**^				**GMFCS level**^**7**^			**CP distribution**^**8**^	
**Characteristics**			Mean dif.^5^	p^6^	95% CI	I	II	III	Unilateral	Bilateral
Number of participants	48	32				29	15	4	27	21
Age, mean (SD)	20 (3)	22 (5)		0.1		19 (2)	21 (3)	19 (2)	19 (2)	21 (3)
Gender, number of males/females	23/25	18/14		0.5		13/16	8/7	2/2	11/16	12/9
**Physical activity mean (SD)**										
% Physical activities^1^	8.6 (3.0)	12.0 (3.9)	-3.4	<0.01*	-5.2 – -2.1	9.2 (3.2)	8.3 (2.4)	5.1 (1.0)	8.8 (2.9)	8.2 (3.2)
% Walking	4.3 (2.3)	8.5 (3.5)	-4.2	<0.01*	-5.6 – -3.0	4.9 (2.3)	4.0 (2.0)	1.5 (1.4)	4.7 (2.2)	3.8 (2.5)
% Running	0 (0.1)	0.1 (0.5)	-0.08	0.18	-0.3 – 0.1	0 (0.1)	0 (0)	0.1 (0.1)	0 (0.1)	0 (0.1)
% Cycling	1.1 (1.2)	1.4 (1.8)	-0.3	0.10	-1.2 – 0.1	1.2 (1.2)	0.9 (1.1)	0.9 (1.4)	1.2 (1.2)	0.9 (1.1)
% Non-cyclic movement	3.2 (1.7)	1.8 (1.1)	1.4	<0.01*	0.7 – 2.1	3.2 (1.7)	3.4 (1.9)	2.6 (1.7)	2.9 (1.6)	3.5 (1.9)
Motility physical activities (g)^2^	43.9 (8.1)					45.5 (5.5)	41.9 (11.0)	40.3 (10.2)	45.8 (5.7)	41.5 (9.9)
Motility walking (g)^2^	52.6 (8.9)					52.9 (6.9)	52.1 (9.5)	52.3 (16.3)	53.8 (7.5)	50.9 (9.9)
0-10 sec walking bouts	124.6 (45.4)					137.0 (41.1)	117.0 (41.9)	63.6 (40.1)	132.0 (39.9)	115.2 (51.0)
10-60 sec walking bouts	88.8 (48.3)					100.8 (50.1)	79.5 (37.5)	36.2 (32.8)	96.1 (45.2)	79.4 (51.6)
1-10 min walking bouts	7.1 (6.0)					7.7 (5.8)	7.7 (6.3)	0.8 (1.5)	7.5 (4.9)	6.6 (7.2)
10-30 min walking bouts	0.1 (0.3)					0.1 (0.3)	0.1 (0.3)	0	0.1 (0.3)	0.02 (0.1)
>30 min walking bouts	0					0	0	0	0	0
**Sedentary time mean (SD)**										
% Sedentary time (sitting + lying)^3^	79.5 (7.1)	74.0 (7.5)	5.5	<0.01*	1.6 – 8.3	77.9 (7.0)	80.6 (5.8)	87.4 (7.6)	78.7 (6.8)	80.6 (7.5)
% Sitting	36.8 (8.1)	36.7 (7.0)	0.1	0.79	-3.1 – 4.1	36.6 (7.6)	35.7 (8.8)	42.3 (8.0)	36.9 (8.6)	36.7 (7.5)
0-10 sec sitting bouts	18.0 (12.2)					15.6 (7.8)	22.0 (17.4)	19.8 (15.2)	14.8 (7.4)	22.0 (15.8)
10-60 sec sitting bouts	34.7 (19.3)					31.7 (14.7)	38.5 (22.8)	41.7 (33.9)	29.6 (15.0)	41.2 (22.7)
1-10 min sitting bouts	40.2 (19.2)					39.7 (18.2)	40.0 (18.2)	44.5 (33.6)	38.1 (18.3)	42.9 (20.4)
10-30 min sitting bouts	11.4 (3.3)					11.8 (3.0)	10.9 (3.3)	10.4 (6.0)	11.8 (3.2)	10.9 (3.5)
>30 min sitting bouts	3.5 (1.9)					3.3 (1.9)	3.3 (1.5)	5.0 (3.0)	3.5 (2.0)	3.4 (1.9)
% Standing	11.9 (5.4)	13.2 (5.3)	-1.3	0.54	-3.2 – 1.7	12.9 (5.3)	11.1 (4.9)	7.6 (6.7)	12.5 (5.5)	11.2 (5.4)
**Self-reported physical activity**										
PASIPD (MET-hr/day), mean (SD)	13.0 (8.6)					15.3 (9.5)	9.8 (6.0)	7.8 (2.4)	14.6 (8.5)	10.9 (8.5)

^1^Physical activities is total duration of walking, running, cycling, and non-cyclic movement, as a % of 24 hours.

^2^g = gravitational forces*100.

^3^Sedentary time is total duration of lying and sitting, as % of 24 hours.

^4^Note that data on motility, bouts and self-reported physical activity were not available for able-bodied controls.

^5^Mean difference between all participants with CP and able-bodied persons.

^6^Difference in characteristics between all persons with CP and able-bodied persons was tested. Difference in physical activity and sedentary time was analysed with regression analyses correcting for age and gender.

^7^Corrected for age and gender, no significant differences were found in physical behaviour between participants with GMFCS level I and II (p > 0.05). Differences with the subgroup GMFCS level III were not assessed because sample size was limited.

^8^Corrected for age and gender, participants with bilateral CP had significantly higher number of sitting bouts 0–10 sec (p = 0.04) and 10–60 sec (p = 0.02) compared to unilateral CP. No other significant differences were found in physical behaviour between participants with bilateral and unilateral CP (p > 0.05).

*indicates significant difference p < 0.05.

GMFCS = Gross Motor Functioning Classification System.

PASIPD = Physical Activity Scale for Individuals with Physical Disabilities.

CI = confidence interval.

Mean dif. = mean difference.

Between participants with GMFCS levels I and II, no significant differences were found in physical activity, sedentary behaviour, and self-reported physical activity level. Since the sample size of the subgroup with GMFCS level III was limited to four persons, statistics were not performed for this subgroup. When comparing unilateral and bilateral participants, only the number of sitting bouts 0–10 sec (p = 0.04) and 10–60 sec (p = 0.02) were significantly higher for participants with bilateral CP.

## Discussion

This was the first study to assess both physical activity and sedentary behaviour in a sample of ambulatory persons with spastic CP after childhood. Persons with CP participated 48 minutes less in physical activities and spent 80 minutes more sedentary per 24 hours, compared to able-bodied controls. A comparison between the present data and guidelines for healthy physical behaviour is difficult. The latter are primarily based on self-report using questionnaires to estimate overall physical behaviour, whereas our data are objective and based on continuous registrations [17]. Future studies defining guidelines based on objectively measured data are necessary.

Consistent with previously published studies, physical behaviour did not differ between participants with GMFCS levels I and II [8,18,19]. However, studies that included GMFCS level III and IV have shown significant associations between GMFCS level and physical activity [8,20,21]. Although we did not test for significance, physical activities seemed to be lower in persons with GMFCS level III (5.1%) compared to GMFCS levels I and II (8.9%), and sedentary times were higher as well (87.4% vs. 78.8%). Therefore, the subgroup of GMFCS III seems to have even less favourable physical behaviour. Since this subgroup was only small, further research is necessary.

Compared to persons with unilateral CP, persons with bilateral CP had significantly more short sitting bouts of 0–10 seconds and 10–60 seconds. Previous studies suggest that these short sitting bouts are favourable behaviour in terms of reducing cardiovascular risk because they break up sedentary time [22]. If these short sitting bouts break up sedentary time, this would also lead to less sittings bouts of more than 30 minutes [23]. However, the numbers of these long sitting bouts were comparable in both subgroups. Since the number of short sitting bouts was the only difference between persons with unilateral CP and bilateral CP, we can conclude that these subgroups are comparable with regard to movement behaviour and health problems.

Although physical behaviour was found to be less favourable in adolescents and young adults with CP, physical strain may be comparable or higher compared to able-bodied persons. Previously, it had been reported that physical strain during walking is higher in persons with CP compared to reference groups [19], and that the physical strain of walking is inversely related to the total time of daily walking [19,24]. Because of higher physical strain, persons with CP may be less active in daily life to conserve energy or prevent fatigue [19,24]. Unfortunately, physical strain during the objective measurement of physical activities was not assessed in the present study. It is unknown how this higher strain in persons with CP relates to the risk of cardiovascular disease and other chronic diseases related to physical behaviour.

Self-reported physical activity was relatively high in the present sample of ambulatory adolescents and young adults with CP (13.0 MET-hour/day, SD = 8.6), as compared to previously published self-reported physical activity in ambulatory persons with CP and meningomyelocele (11.3 MET-hour/day (SD = 8.6)) [17]. However, objectively measured physical activity was also reported to be lower in those groups: 8.1% in adults with bilateral CP and 7.8% in ambulatory persons with meningomyelocele, compared to 8.6% in the current sample [25].

### Limitations

Although a large number of persons with varying GMFCS levels were invited to participate, our sample included only four persons with GMFCS level III. Further research is required in persons with lower functioning GMFCS levels, including GMFCS level III and IV and wheelchair-bound persons. Wearing the activity monitor may have influenced activities in daily life, despite participants’ reports that they were able to perform their regular activities. Although all measurements took place on weekdays and no significant differences were found between days, measurement duration in participants with CP was one to three days while measurement duration in able-bodied persons was two days. Furthermore, comparisons between persons with CP and able-bodied persons have to be interpreted with some caution since in able-bodied persons a previous version of the activity monitor and software was used. However, the underlying technique and analysis procedures were comparable between versions and therefore no differences between systems versions are expected.

Our study may overestimate physical activity because of selection bias; persons with CP interested in physical activity and sports may have been more likely to participate in the study. Despite of that, this group was less physical active and had more sedentary time compared to reference.

## Conclusions

Objective measurements show that ambulatory adolescents and young adults with CP are less physically active and spend more time sedentary compared to able-bodied persons, suggesting that this group may be at increased risk for health problems related to less favourable physical behaviour.

## Consent

Consent for publication has been obtained from the person on Figure 1.

## Abbreviations

CP: Cerebral palsy; GMFCS: Gross Motor Functioning Classification System; MET: Metabolic equivalent; PASIPD: Physical Activity Scale for Individuals with Physical Disabilities; g: Gravitational forces.

## Competing interests

The authors declare that they have no competing interests.

## Authors’ contributions

CN contributed to design, data analysis, interpretation of data and drafting the manuscript, JS to design, acquisition, interpretation of data and reviewing the manuscript, HS and MR to design, interpretation of data and reviewing the manuscript, and RB to design, data analysis, interpretation of data and drafting the manuscript. All authors read and approved the final manuscript.
